# An Historical View of Surgery in the Sixteenth Century

**Published:** 1799-09

**Authors:** 


### *An Hiſtorical View of Surgery in the Sixteenth Century.*

[Continued from our last Number, pp. 54—60.]

§. 7. THE diſcovery of the high operation was the work of neceſſity and accident. PETER FRANCO, of Turrieres, in Provence, ſurgeon at Berne, Lauſanne, and Orange, was requeſted in the year 1560 to perform this operation for lithotomy, at Lauſanne, on a child two years of age. He had already begun to operate with the ſmall apparatus, when he found that the ſtone was of the ſize of a hen's egg, and conſequently too large to be be removed in that manner. The child's parents infifted that the operation fhould neverthelefs be finifhed; and as the bladder very much projected above the *offa pubis*, he determined upon making the incifion above thefe bones. Although he eventually fucceeded in this bold attempt, yet he prudently diffuades his brethren from imitating that practice: and indeed the danger to be apprehended from the effufion of the urine into the abdomen is fo great, that even the improvements made by DOUGLAS, on the high apparatus of Franco, have not much diminifhed it.—In order to remove the ftone from female patients, Franco rejects both the large and fmaller apparatus, while he propofes merely the dilatation of the urethra, by means of an inftrument invented by himfelf; after which he extracts the ftone with the forceps, without diffecting the parts. He likewife invented a gorgeret, and a forceps, the arms of which expand in the bladder; but it muft be acknowledged, that the ufe of thefe inftruments is extremely inconvenient.

§. 8. A very painful and unneceffary operation excited great attention during this century, although it had been previoufly performed. The reader will perhaps fmile at an attempt to repair and reftore that prominent part of the human face, the nofe, when mutilated by accident.—BARRI, an Italian anthor, in his "*Italia illuftrata,*" page 1060, fol. Francof. 1600, confiders VINCENT. VIANEO as the inventor of this fingular practice.

Two Sicilian furgeons of the name of BRANCA, father and fon, had fo early as the latter end of the fifteenth century, acquired celebrity by the fuccefsful renovation of nofes; an art which became hereditary in the family of the BOJANI. But CASPAR TAGLIACOZZI, Profeffor at Bologna, raifed this art to fuch high perfection, as to render it one of the principal branches of furgery: he became fo celebrated by his operations, that his cotemporaries erected a public monument at Bologna, where he is reprefented with a nofe in his hand. This operation is defcribed in an interefting work, intitled, "*Tagliacot. de curtor. chirurgia*;" fol. Venet. 1597; in which he compares it (p. 47) to the ingrafting of trees, expatiates on the dignity and ornament of the nofe, and endeavours to prove that there is not the leaft danger in cutting out a piece from the biceps mufcle of the arm. With refpect to the diet to be obferved during the operation, he gives ample and rigid inftructions, while he maintains that the inoculated nofe is poffeffed of a more acute fmell, and that it generally grows much larger and ftronger than the organ which had been accidentally loft. The hair on the new nofe, according to his account, in many inftances grows fo faft that it muft be regularly fhaved.

Two refpectable eye-witneffes, FORTUN. LICETI, in his book, "*De Monftris*," lib. ii, cap. 29, p. 108, and JOH. BAPT. CORTESI, as quoted by HALLER, in his "*Biblioth. Chirurg.*" Vol. I. p. 293, confirm the truth of Tagliocozzi's affertions, that he has fuccefsfully reftored not only nofes, but likewife ears and lips. MARC. ANT. ULMO, in his "*Phyfiol. barbæ human.*" p. 230, fol. Venet. 1604; and RANCHIN, in his "*Quef-tiones en Chirurg.*" p. 118, both fpeak in favour of this operation:—VESALIUS alfo, in his "*Chirurg. magn.*" lib. iii. cap. 9, p. 983, defcribes the whole procefs of operating, with all the minutiæ, as if he actually had performed it himfelf. In the "*Oeuvres d'Ambr. Paré*," liv. xxiii. cap. 2. p. 574, this illuftrious writer relates the cafe of a knight, le Cadet de ST. THOAN, who having loft his nofe by a cut, had another fuccefsfully engrafted by this artificial procefs. FABR. VON HILDEN, in his "*Centur.* III." Obferv. xxxi, p. 214, fol. Francof. 1646, alfo mentions a remarkable cafe of a lady, who recovered her nofe by means of this operation, performed in the year 1592, by GRIFFON, a furgeon at Laufanne.

§. 9. It will be neceffary for the elucidation of chirurgical hiftory, to take a fhort view of the general ftate of furgery in the fixteenth century, and to give fome account of the violent difputes which prevailed in France, on the prerogatives of the medical art over thofe of furgery, but particularly on the contefted privileges of the furgeons. Although the documents relative to this fubject have been partly printed, or have at leaft not been withheld from the infpection of hiftorians, yet no part of medical hiftory has been conducted with more partiality, and lefs regard to truth, by both parties, than that which is now under confideration. The author of the work entitled "*Recherches fur l'origine et le progrès de la Chirurgie en France*" is guilty of the groffeft mifreprefentation, though this book has by fome been afcribed to FRANC. QUESNAY. He is fo much attached to party-intereft, that his account refembles more a judicial plea than a faithful relation of facts.—PASQUIER, in his "*Recherches de la France*," fol. *Paris*, 1620, deferves much more credit, and for this reafon, the moft important points relative to that extraordinary difpute have been briefly collected from his more authentic ftatement.

The furgeons of Paris have, fince the time of LANFRANCHI formed a diftinct body, called the College of St. Côme; they obtained additional and refpectable privileges of PHILIP, furnamed the Fair, in 1311, which entitled them to equal rank with the members of the medical faculty; hence they could not bear the idea that barbers fhould ufurp the right of bleeding, applying plafters, and treating external injuries and ulcers. In confequence of this encroachencroachment, the ſurgeons, in 1425, obtained an act or arrêt of the Parliament of Paris, by which the performance of chirurgical operations was prohibited to the barbers, while they were permitted to dreſs wounds, and extirpate corns by the knife. But the phyſicians embraced the cauſe of the barbers, and inſtructed them in the practice of ſurgery, with a view to take vengeance on the ſurgeons, who, it was affirmed, had uſurped medical privileges. The complaint of grievances which the ſurgeons, on this occaſion, laid before the faculty, in the years 1491 and 1494, were not attended with any other effect, than the promiſe to change the ſituation of affairs; neverthele ſs, the members of the faculty were permitted to deliver anatomical lectures to barbers, in the French language. The ſurgeons again, though in vain, repreſented to the faculty, that they acted contrary to the laws made by themſelves, by permitting their members to inſtruct barbers in the knowledge of anatomy, and this in their native language. However, no other redreſs could be obtained, but that of licenſing the ſurgeons to undertake public diſſections, and of granting them a certain rank above the barbers, for which they paid ſixty *ſolidos* annually to the treaſurer of the faculty. This event took place in the year 1502; and in 1505 the ſurgeons renewed their application in the character of ſcholars or pupils to the faculty, whom they intreated to confirm their privileges; but HELIN, the ſenior of that body, ſent them the diſcouraging anſwer, that their pretended rights or immunities had been acquired by ſurreptitious means.

§ 10. In the ſame year, the phyſicians of Paris, as Paſquier expreſſes himſelf, paſſed the *Rubicon,* and entered into a formal contract with the barbers, who, on account of their implicit obedience, were patronized in preference to the ſurgeons. The barbers were conſequently, in contempt of the ſurgeons, pronounced to be the true ſcholars of the faculty: they were matriculated under that name; but a promiſe was exacted from them, according to which they were not allowed to adminiſter internal medicines, without conſulting, in every caſe, a member of the medical faculty; they farther agreed to undergo an examination, previous to their commencing buſineſs as maſters. Since that period, the barbers have been no longer called *barbitonſores,* the complaiſant faculty having conferred on them the more honourable title of *Chirurgici a Tonſtrina,* or *Tonſores Chirurgici.* A few days after this change, the faculty proceeded to ſuch extremities as to proſecute the ſurgeons in a court of law, becauſe they had received information, that ſeveral ſurgeons had preſcribed internal remedies, without the previous advice of a phyſician.

Probably, in that age, no man of genius and activity preſided over the College de St. Côme; for no ſooner was STEPHEN BARAT elected preſident of of that college, than the ſituation of affairs was thoroughly changed. In the year 1515, he urged the faculty to exempt the ſociety of ſurgeons from the oppreſſive tax they were obliged to pay annually, and not to compel them to attend the lectures given by members of the faculty. As Barat addreſſed himſelf to the whole univerſity, and as old Helin, the moſt zealous antagoniſt of the ſurgeons, died in the ſame year, this manly remonſtrance had the deſired effect. The univerſity iſſued a decree, by which the ſurgeons of Paris were nominated *Scholaſtici*, or perpetual ſcholars of the faculty. But ſtill greater immunities were granted to the ſurgeons in 1545, by the good offices of WILLIAM VAVASSEUR, principal ſurgeon at the court of FRANCIS I. He ſuccefsfully effected a complete ſeparation of the barbers from the ſurgeons, and at the ſame time obtained a decree, in conformity to which every maſter of the chirurgical art, if he wiſhed to obtain the privilege of exerciſing his profeſſion, was obliged to ſtudy the Latin language, logic, and other elementary ſciences. By this favourable regulation, the College of Surgeons was at once raiſed to the rank of a learned ſchool, and obtained at length the right of creating Maſters, Bachelors, Licentiates, and Doctors of Surgery. In conſequence of this arrangement, HENRY II. granted to the members of the Chirurgical College of St. Louis, all the prerogatives attached to a faculty; and the patent iſſued on that occaſion, was regiſtered in the parliamentary laws, under the name of *Lettres d'Octroi.*

§ 11. In the year 1551, the medical faculty, under the deanery of JOHN DU HAMEL, recommenced the diſpute againſt the ſurgeons. Although RUDOLPH LE FORT, dean of the College of St. Louis, zealouſly defended the ſurgeons, yet Du Hamel found the means of procuring a repeal of the decree enacted in 1515; and, contrary to the ſpirit of that law, the ſurgeons were again obliged to ſubmit to an examination before the medical faculty. Under HENRY III, however, the ſurgeons once more obtained a confirmation of their privileges, in 1577, by virtue of which they were entitled to confer academical dignities; and, notwithſtanding the new oppoſition of the faculty in 1579, the ſurgeons, as well as the univerſity of Paris, were in the ſame year favoured with an indult of Pope GREGORY XIII, while DE THOU vindicated the cauſe of the former, in a ſpirited and ſuccefsful manner, againſt the oppreſſions of the faculty. The colleges ſubſequently eſtabliſhed by ſurgeons, acquired ſuch a degree of authority, that, in the year 1596, they were empowered to give ſerious orders to the barbers, in difficult chirurgical caſes always to conſult a ſworn ſurgeon, and upon no account to undertake the treatment of any other but the ſlighteſt external injuries. Theſe privileges and prerogatives of the ſurgeons of Paris

Paris were farther confirmed by HENRY the Great, in 1602; and LOUIS XIII, in 1614.

[*In our next Number, we shall continue this article, and begin with an account of the most celebrated surgeons of that century, in chronological order.*]

